# Resource Discovery for the Twenty-First Century Library: Case Studies and Perspectives on the Role of IT in User Engagement and Empowerment

**DOI:** 10.5195/jmla.2021.1157

**Published:** 2021-01-01

**Authors:** Dana Haugh

**Affiliations:** 1 dana.haugh@yale.edu, Web Services Librarian, Harvey Cushing/John Hay Whitney Medical Library, Yale University, New Haven, CT

*Resource Discovery for the Twenty-First Century Library: Case Studies and Perspectives on the Role of IT in User Engagement and Empowerment* offers an engaging overview of the discovery landscape through case studies, vendor perspectives, and speculative fiction. Editor Simon McLeish's enthusiasm for the topic is palpable throughout the monograph and adds another layer of engagement for readers.

The book begins by outlining the current landscape, positioning the reader firmly in the “now” of discovery, which is helpful for those outside the resource discovery world. The foreword and introduction give high-level summaries of the role of discovery layers, mainstream search interfaces, and the complexities of creating a system that both meets and anticipates the needs of its users.

Libraries are moving beyond locally acquired collections toward facilitating access to resources that are not yet licensed or owned, and discovery layers have become even more essential to finding a wide array of content. As Lorcan Dempsey points out in the foreword, “the acquired library collection is now actually only one resource among many of potential interest” (p. xxiii). Historically, library collections have driven discovery because users could find only what was already available; now discovery is starting to drive collections. Anything that may be of use to a researcher is reason enough to index it, and search results are starting to include records of unlicensed materials, free Internet content, library website pages, research guides, and librarian specialties (p. xxv).

After establishing the landscape, the book moves into case studies from the Australian National University, the National Library of Singapore, the Institute of Electrical and Electronics Engineers (IEEE), EBSCO Discovery Service, and the University of Oxford. The case study from the University of Oxford is particularly useful to practitioners who are exploring the user experience side of resource discovery because it includes questions for conducting patron interviews. The juxtaposition of library case studies and vendor case studies was an interesting editorial choice. It is difficult to read the vendor case studies without seeing a sales pitch; however, both are well written and informative, if somewhat self-serving.

The book moves on to confront the challenges of discovery via mainstream search engines, such as Google, and offers suggestions for how to optimize resource descriptions to ensure they are more visible to users who are looking for them. Resource discovery using Blacklight and linked data is covered in the next two chapters, and chapter 11 rounds out the book with a somewhat redundant recap of what is known about discovery and what the future may hold.

The final chapter, however, deviates from the known and enters the speculative world of science fiction. Perhaps the most intriguing chapter in the book, chapter 12 begins with a poignant quote from Denis Diderot foreshadowing a time when the multitude of information will make uncovering the truth difficult, and something will have to be done to facilitate its discovery (p. 177). Authors Wolfram Horstmann, David De Roure, and Simon McLeish offer short vignettes of what the future of research and scholarship may look like: from a completely open access, collaborative future to one monopolized by a single publisher hoarding the world's scholarship. The authors offer this chapter as a way “to inspire interest in and thought about the future of discovery” (p. 179). It is a memorable and entertaining way to end the book, and the vignettes will stick with the reader long after.

Overall, the title is well researched and expertly builds on and pushes forward the scholarship in the field. One of the book's shortcomings is its figures. About 40% of the book's figures are nearly unreadable due to extremely poor contrast or miniscule type. One chapter includes 10 figures of varying readability, most of which add little value. Another chapter uses 15 figures and tables across its 18 pages, which feels more like an attempt to fill space rather than illustrate the case study. Overall, the monograph would have been stronger if all figures were of higher resolution and contrast, and authors were limited in how many they could include.

As with any text covering present-day technologies, the timeliness of the topic does not necessarily guarantee its timelessness. Some of the social media platforms mentioned in chapter 8 and insights of this book do prevail and prove useful to those working in all aspects of librarianship.

